# ROCA – An ArcGIS toolbox for road alignment identification and horizontal curve radii computation

**DOI:** 10.1371/journal.pone.0208407

**Published:** 2018-12-26

**Authors:** Michal Bíl, Richard Andrášik, Jiří Sedoník, Vojtěch Cícha

**Affiliations:** CDV–Transport Research Centre, Brno, Czech Republic; University of Louisville, UNITED STATES

## Abstract

We present the ROCA (ROad Curvature Analyst) software, in the form of an ESRI ArcGIS Toolbox, intended for vector line data processing. The software segments road network data into tangents and horizontal curves. Horizontal curve radii and azimuth of tangents are then automatically computed. Simultaneously, additional frequently used road section characteristics are calculated, such as the sinuosity of a road section (detour ratio), the number of turns along an individual road section and the average cumulative angle for a road section. The identification of curves is based on the naïve Bayes classifier and users are allowed to prepare their own training data files. We applied ROCA software to secondary roads within the Czech road network (9,980 km). The data processing took less than ten minutes. Approximately 43% of the road network in question consists of 42,752 horizontal curves. The ROCA software outperforms other existing automatic methods by 26% with respect to the percentage of correctly identified curves. The segmented secondary roads within the Czech road network can be viewed on the roca.cdvgis.cz/czechia web-map application. We combined data on road geometry with road crashes database to develop the crash modification factors for horizontal curves with various radii. We determined that horizontal curves with radii of 50 m are approximately 3.7 times more hazardous than horizontal curves with radii accounting for 1000 m. ROCA software can be freely downloaded for noncommercial use from https://roca.cdvinfo.cz/ website.

## Introduction

Although road alignment is used in many traffic safety-related studies, information concerning whether a road segment is a curve or a tangent is often missing in the original road network databases. Identification of horizontal curves from road network data is still a time-consuming and error-prone process. Moreover, when derived manually, such work cannot be reproduced in the future. A large amount of road network data is currently stored in GIS, with a sufficient spatial precision allowing for automated data processing. A demand for a fully automated tool for road alignment extraction from digital data therefore exists (e.g., [1, 2, 3]).

A lack of data on road alignment and a need to identify road geometry manually is in all probability behind the relatively low number of individual horizontal curves used in the majority of the studies. McBean [4] had, for example, only 100 horizontal curves when working with data from Great Britain, as well as Othman at al. [5] when studying the safety performance of horizontal curves from naturalistic driving data. Fitzpatrick et al. [6] worked with 260 curves. Fink and Krammes [7] and Persaud et al. [8] used a higher number of data, more than 500. In contrast, Zegeer et al. [9], Sakshaug [10] and Khan et al. [11] had more than 10,000 curves. They obtained the information about road geometry from national road databases. The majority of national road databases do not usually contain such information for the entire network.

### Previous work on automated identification of horizontal curves from digital data

There is no fully automatic tool able to precisely identify horizontal curves and tangents from digital vector data in existence at present. Semi-automated identification is a frequently used approach [12, 13]. An expert determines the endpoints of a horizontal curve on a screen and its radius and length are then automatically calculated. This approach is not suitable, however, for large data sets. The “Curve Calculator” [14] is a typical representative of these methods.

Xu and Wei [3] presented a method based on azimuth computation for each road vertex. They argue that working with GIS is the most effective way from both time and economic perspectives, when analyzing large datasets. Li et al. [1] have developed a fully automated method for a GIS which computes the "bearing angle". They applied a threshold when deciding between horizontal curves and tangents. Li et al. [1] stated that *“In addition to this semiautomatic approach*, *no literature documenting a fully automatic method was found*. *Therefore*, *CurveFinder is truly innovative and unique by offering the means to automatically obtain curve location and geometric information from GIS roadway maps*.*”* We will present here an approach which outperforms CurveFinder, because our approach uses, not only the bearing angle to decide if a road vertex belongs to a horizontal curve or to a tangent, but five more explanatory variables (EVs) of road geometry and a classification procedure instead of a simple threshold (see the Methods section below for details).

### Crash modification factors for horizontal curves in various countries

Crash modification factors (CMFs) for horizontal curves are among the most applied fields where road geometry is being used. We selected this application to demonstrate the performance of the ROCA software within traffic safety related studies.

Jurewicz and Pyta [15] worked with horizontal curves previously categorized into three groups. They concluded that if the number of crashes in curves with a radius of more than 1500 m is set equal to 1, the corresponding values were 1.422 for curves with a radius between 600 and 1500 m and 2.437 for curves with a radius less than 600 m. Persaud et al. [8] used data on horizontal curves with radii between 87 and 1150 m in their research on identification of hazardous highway curves. They found the 15 worst curves with the highest risk based on computation of the Empirical Bayes Estimate. Elvik [16] presented a metastudy on CMFs based on data from several previously published studies from various countries. Crash rates on curves, in relation to the length of the preceding tangent, are also a focus of research (e.g., [17, 18]), as well as crash rate differences between horizontal curves and tangents (e.g., [19, 20]).

The aim of this work is to present the ROCA software which is able to substantially increase the efficiency of the road safety-related research where information on geometry of road segments is required.

## Data and methods

### Data

Sections of the Czech road network were provided by the Road and Motorway Directorate of the Czech Republic in the form of spatial (GIS) data. We focused on secondary roads which are intended to provide a service within different regions of the Czech Republic. An expert manually identified the road geometry for 52 randomly selected secondary roads. We split these roads into two groups: a training set consisting of 32 roads (2,385 vertices, 68.6 km) and a validation set consisting of 20 roads (3,360 vertices, 74.1 km).

We also worked with traffic crashes which occurred on secondary roads within the Czech road network over the period 2009–2016. These data come from the Police of the Czech Republic, Traffic Crash Database. As of 2009, data have been geo-localized by the use of GPS. All traffic crashes, excluding intersections and streets in urban areas, were used in the analyses. In total, we considered 56,710 traffic crashes.

### Methods

This work stems from a classification approach for effective identification of the road geometry introduced by Andrášik and Bíl [2]. More specifically, we applied the same idea of using a classification method to determine horizontal curves and tangents within a road network, but selected a different classification approach to allow for full automation of the entire process (construction of toolbox in ArcGIS).

A classification tree [21] was originally applied in Andrášik and Bíl [2] being the best approach among the considered classifiers (classification tree, neural network, multiple logistic regression and a simple threshold for circumscribed and osculating circles as a benchmark). The classification tree approach has several advantages: simple application, automatic choice of relevant EVs and a straightforward interpretation [22]. Drawbacks were also reported [22], however, the most important being was the common overfitting problem which required further pruning of a tree and thus made the entire process complicated in terms of automated data processing. We therefore substituted the classification tree method with a naïve Bayes classifier [23]. The naïve Bayes classifier does not suffer from overfitting [24] and even works well with very small training datasets [25]. It is consequently an appropriate approach for automated use in our software. Furthermore, the naïve Bayes classifier has a low memory and computational time requirements [26].

### Naïve Bayes classifier

The naïve Bayes classifier is based on applying Bayes’ theorem and assuming independence between the explanatory variables. Although this assumption is not realistic in most cases, as well as in our case, the performance of the naïve Bayes classifier is comparable with more sophisticated state-of-the-art classifiers [24, 26]. We only mention the main ideas of the naïve Bayes classifier. For the theoretical details, see Dougherty [23].

According to the Bayes theorem, it holds that

$$P(h.curve|EVs)=\frac{f(EVs|h.curve)P(h.curve)}{Z},$$ (1)

$$P(tangent|EVs)=\frac{f(EVs|tangent)P(tangent)}{Z},$$ (2)

where *f*(*EVs*|*h*.*curve*) and *f*(*EVs*|*tangent*) are joint probability density functions of EVs for vertices belonging to horizontal curves and tangents, respectively, *P*(*h*.*curve*) and *P*(*tangent*) are prior probabilities of observing a horizontal curve and tangent, respectively, when randomly choosing a vertex (a point; a set of vertices forms a line in a GIS), and *Z* stands for a normalizing constant.

The prior probabilities can be set either both as 0.5, or according to relative frequencies of vertices belonging to horizontal curves and tangents, respectively. Since the normalizing constant is the same for both *P*(*h*.*curve*|*EVs*) and *P*(*tangent*|*EVs*), there is no need to evaluate it. If *P*(*h*.*curve*|*EVs*) is greater than *P*(*tangent*|*EVs*), we classify the vertex as belonging to a horizontal curve. Otherwise, the vertex is classified as belonging to a tangent.

The independence assumption leads to the following simplification of the joint probability density functions:

$$f(EVs|h.curve)=\prod_{i=1}^{n}f({EV}_{i}|h.curve),$$ (3)

$$f(EVs|tangent)=\prod_{i=1}^{n}f({EV}_{i}|tangent),$$ (4)

where *n* is the total number of EVs.

It is much easier to estimate several univariate probability density functions than trying to estimate the joint probability density function. Furthermore, it allows us to avoid the curse of dimensionality. These univariate probability density functions have to be estimated from a training dataset. John and Langley [27] demonstrated that the performance of the naïve Bayes classifier can be significantly improved by the use of the kernel density estimation (a non-parametric method for estimating a probability density function) when compared to a mixed normality assumption and the use of Gaussian distributions. We consequently applied the kernel approach to estimate the univariate probability density functions.

We used the same six EVs as introduced in Andrášik and Bíl [2]:

- An angle between three consecutive points (sometimes called “the bearing angle” [1],
- a cumulative angle at three points,
- a cumulative angle at five points (a similar characteristic as the direction change used by Xu and Wei [3])
- a radius of a circumscribed circle,
- a radius of an osculating circle,
- the distance between two consecutive points.

**Fig 1:**
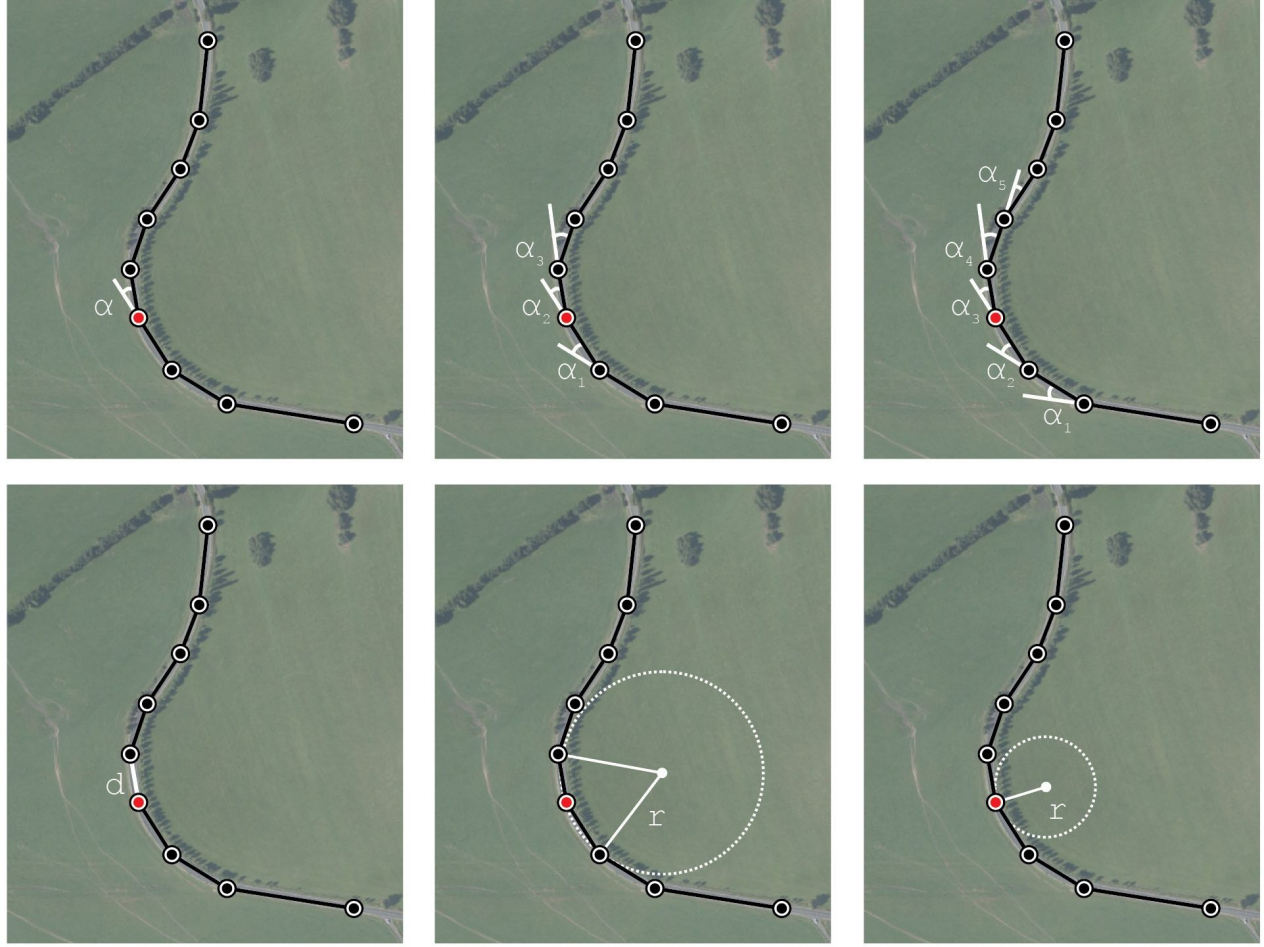
Graphical explanation of EVs.

These EVs are graphically explained in Fig 1. See Table 1 in Andrášik and Bíl [2], for details on the calculations of the EVs. The selected EVs are frequently used for curve identification [28]. Specifically, an angle between three consecutive points [1], a cumulative angle at free and five points and a radius of a circumscribed circle [29] are suitable characteristics of a road curvature. The radius of an osculating circle was also selected as it clearly differentiates between horizontal curves and tangents [2]. We also considered the distances between consecutive points in order to take into account the density of the points.

**Table 1:** Descriptive data for the identified horizontal curves. Two choices of “Maximal radius of curve” were selected.

Maximal radius of curve	2100 m	1000 m
Number of curves	42,752	39,943
Total length of curves	4,314 km	3,577
Proportion of the total length of curves	43%	36%
Number of TCs per kilometer at curves	5.9	6.1

### CMF for horizontal curves

We computed CMF for the horizontal curves of secondary roads within the Czech road network in order to demonstrate the capability and one of the potential applications of the ROCA software. Traffic crashes were then joined to the closest homogenous road segments to obtain basic descriptive statistics and input for estimation of the CMF.

We followed the approach of Persaud et al. [8] which was also applied by Elvik [16] for a comparison of CMFs developed in various countries around the world. We assume that the number of traffic crashes (TCs) within a horizontal curve depends on the annual average daily traffic (AADT), the length of the horizontal curve (L [m]), the radius of the horizontal curve (R [m]) and ratio R/L in the following manner:

$$Number\;of\;TCs\;per\;year=e^{\beta_{1}+\beta_{2}(L/R)}{AADT}^{\beta_{3}}L^{\beta_{4}}R^{\beta_{5}}.$$ (5)

Parameters *β*_*i*_, *i* = 1, …, 5, can be fitted by the use of the negative-binomial regression.

In the following step, we calculated crash rates (number of TCs per year per million kilometers of travel) in horizontal curves with radii ranging from *R*_*min*_ = 50 m to *R*_*max*_ = 1000 m to achieve CMF comparable with CMFs presented by Elvik [16]. Since a horizontal curve is usually part of a circle, it can be assumed that the length of a horizontal curve linearly depends on its radius, i. e. *L* = *ωR*, where *ω* > 0 is a deflection angle in radians.

Finally, we calculated the relative crash rates for horizontal curves by establishing the crash rate in horizontal curves with the largest radius (1000 m) as the reference. This means that the relative crash rate for such curves equals one, and other crash rates are recalculated according to this rescaling. In conclusion, the relative crash rate only depends on the varying radius of the horizontal curve:

$$Relative\;crash\;rate={\left(\frac{R}{R_{max}}\right)}^{{(\beta_{4}-1)\beta }_{5}}$$ (6)

We have also computed additional frequent parameters used in evaluation of road sections, such as detour ratio [30], number of turns [31] and the average cumulative angle [32].

## ROCA software description

### Data preparation

Road network have to be separated into individual road sections. Users should initially check the quality of their data geometry, particularly if any original segmentation of data exists. This occurs, for example, when roads cross regional borders. Additional common line geometry data errors (vertex crossing, redundancy of vertices) can distort classification and the identified type of feature geometry. We therefore recommend checking the quality of the input line data and reshaping them in advance in the Editor or Topology toolbar.

### Input data

**Fig 2:**
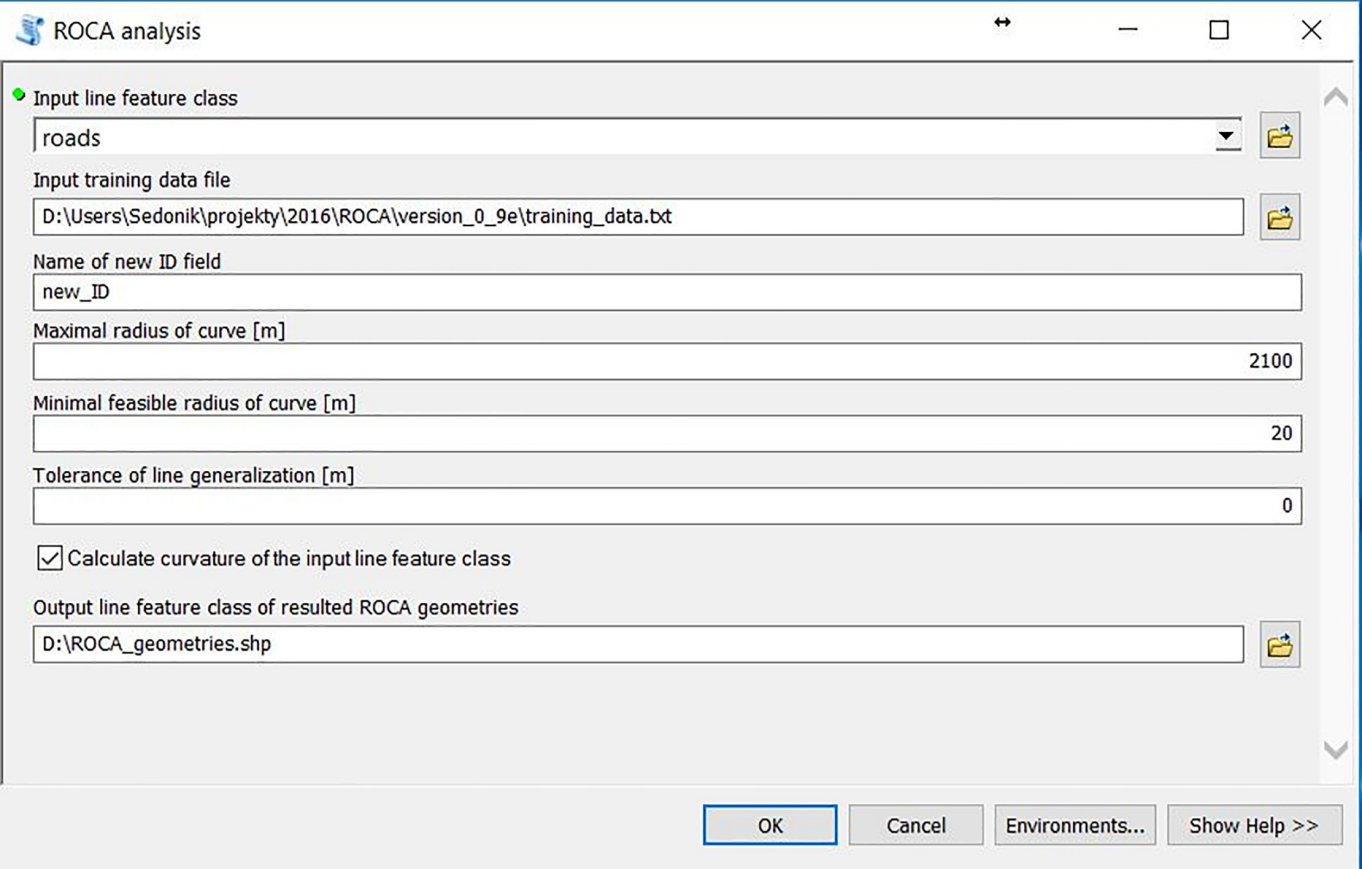
ROCA environment within the ArcGIS software.

Apart from the input line feature class of road network sections, users are allowed to input a predefined or their own text file (Input training data file, see Fig 2) with coordinates of road section vertices and user-defined geometry classification. The structure of a training text file is as follows:

- road section ID                    #
- X coordinate          # coordinates of a polyline vertex
- Y coordinate          #
- geometry classification {0,1} # 0 for a tangent and 1 for a horizontal curve

Users are allowed to define the maximum value of a radius above which a segment is considered a tangent (Maximal radius of curve [m], see Fig 2). Commonly used radii thresholds are usually between 1000 and 2500 m (e.g., [16]).

A value of the minimal accepted radius of a horizontal curve (Minimal feasible radius of curve [m], see Fig 2), can be established in order to identify potential errors in input data. A warning with the number and total length of the curves with a lower radius than predefined here will be shown in a final report message.

**Fig 3:**
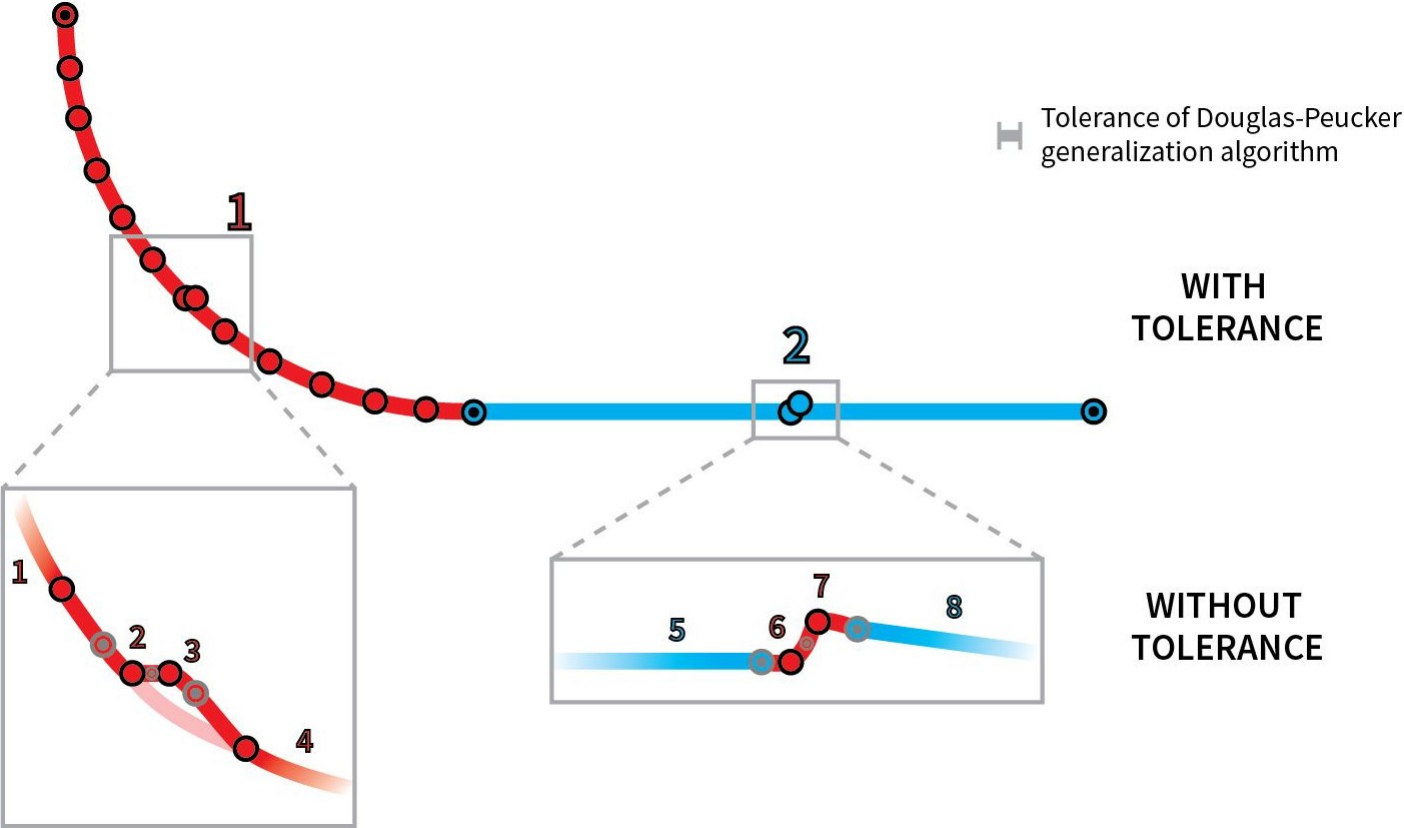
Examples of errors which are usually present in databases which contain manually digitized data. The resulting ROCA geometries are smooth and noiseless when the Douglas-Peucker generalization algorithm (with user-defined tolerance) is applied. The results can also be incorrectly classified in cases when no generalization algorithm is used. The numbers represent individual homogenous road segments.

We strongly recommend the user apply data generalization to filter out possible errors in line data. A tolerance can be set (Tolerance of line generalization [m], see Fig 2). Douglas-Peucker generalization algorithm is embedded inside the ROCA software to simplify the input lines and to remove redundant vertices which could also distort the final classification (see Fig 3).

### Data processing

**Fig 4:**
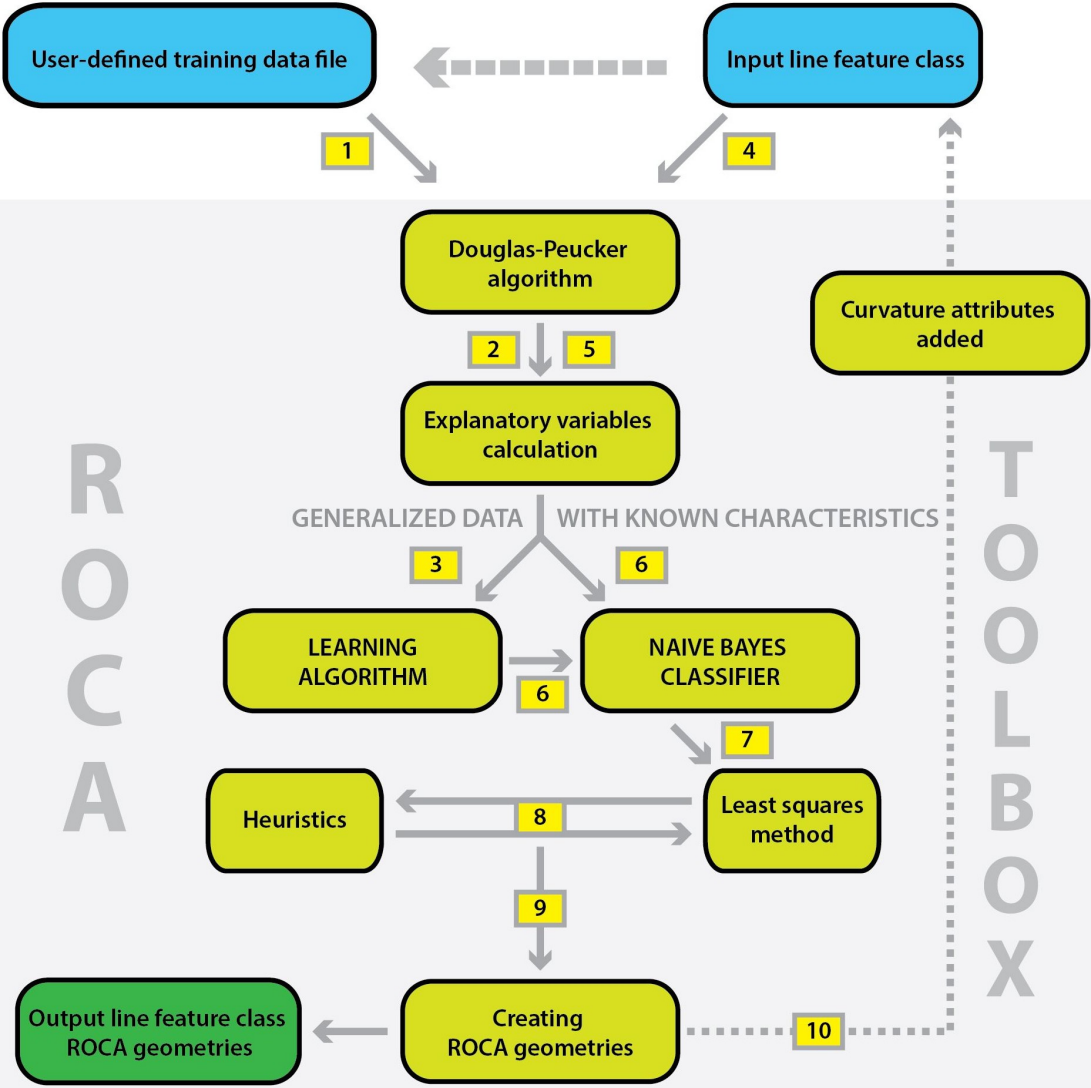
ROCA flow chart.

The overall ROCA process is described in Fig 4. We recommend that users prepare their own training data sets (step 1), otherwise a default training set (based on secondary roads from the Czech road network) will be used. The training data set is then generalized (step 2) and six EVs are computed for each vertex (see the Methods section for more information). The learning algorithm (step 3) is applied to prepare a naïve Bayes classifier for the entire data set (step 4). The generalization is processed and the EVs are computed for all the data. The naïve Bayes classifier is then used for identification of curves and tangents (step 7). The least squares method and heuristics are applied to estimate horizontal curve radii (step 8). Finally, an output file containing individual road alignment geometry is created (step 9) and three new fields with curvature attributes are added to the original line data file (step 10).

### ROCA outputs

The primary ROCA outputs are a new line feature class containing both horizontal curves and tangents. Each output segment is stored as an individual line feature geometry with the following attributes:

- ID of an input line section of the road network,
- type of ROCA geometry (0 –tangent, 1 –curve),
- radius of a horizontal curve,
- X, Y of the center of the horizontal curve,
- azimuth of a tangent (horizontal angle between the tangent and the direction to the north; measured clockwise),
- length of ROCA geometry.

New attributes are also added to the original input sections of the road network:

- detour ratio (sinuosity of the road section) as a ratio of the real (network route, polyline length) and shortest (Euclidean) distance between the endpoints of the road section,
- number of turns along a road section,
- average cumulative angle turned per kilometer of a road section.

The final report, when the ROCA analysis is completed, is written as a message in executing the tool window of the ROCA analysis. It contains:

- the number of tangents with their total length,
- the number of horizontal curves (with defined radii) with their total length,
- the number of horizontal curves with radius lower than the minimal feasible radius of the curve (defined by a user) and their total length; it highlights possible errors in the input line feature class geometry.

The toolbox was programmed in Python 2.7 and can be used within ESRI ArcGIS (10.1–10.5). No installation of the ROCA toolbox is needed, the toolbox can be simply added to ArcToolboxes. Our software can be downloaded from roca.cdvinfo.cz website for free for noncommercial use.

## Results

### Validation of the naïve Bayes classifier

We built the naïve Bayes classifier from our training set consisting of 32 secondary roads (2,385 vertices, 259 curves). First, we calculated the EVs for the entire training set. Next, we estimated the probability density functions of EVs, separately for horizontal curves and for straight segments, with the use of the kernel density estimation. On the basis of these probability density functions, any new road geometry can be classified (see section Naïve Bayes classifier).

We estimated the performance of the ROCA software by using a separate validation set consisting of 20 secondary roads (3,360 vertices). The validation set contained 220 horizontal curves. The success rate (ratio of correctly classified vertices) of the Bayesian classifier was 82.4% which is similar to other classifiers considered in [2]. The majority of the incorrectly classified vertices were located at the ends of horizontal curves.

Rasdorf et al. [29] estimated the success rates of correctly identifying a horizontal curve as 78% for the “Curve calculator”, 69% for the “Curve Finder” and 80% for the “Curvature extension”. Our approach was able to correctly identify 95% horizontal curves from the validation set. It therefore seems that our approach is superior to the automatic “Curve Finder” by 26%, and to the semiautomatic “Curve calculator” and “Curvature extension” by 17% and 15%, respectively.

### Example of ROCA results

ROCA was applied to the secondary roads within the Czech road network (9,980 km) to demonstrate its computational efficiency. The calculation lasted less than ten minutes (9 min 45 s) on a standard PC (4 core Intel i7-2600 3.4 GHz processor, RAM 8GB, Windows 10 Pro 64bit).

**Fig 5:**
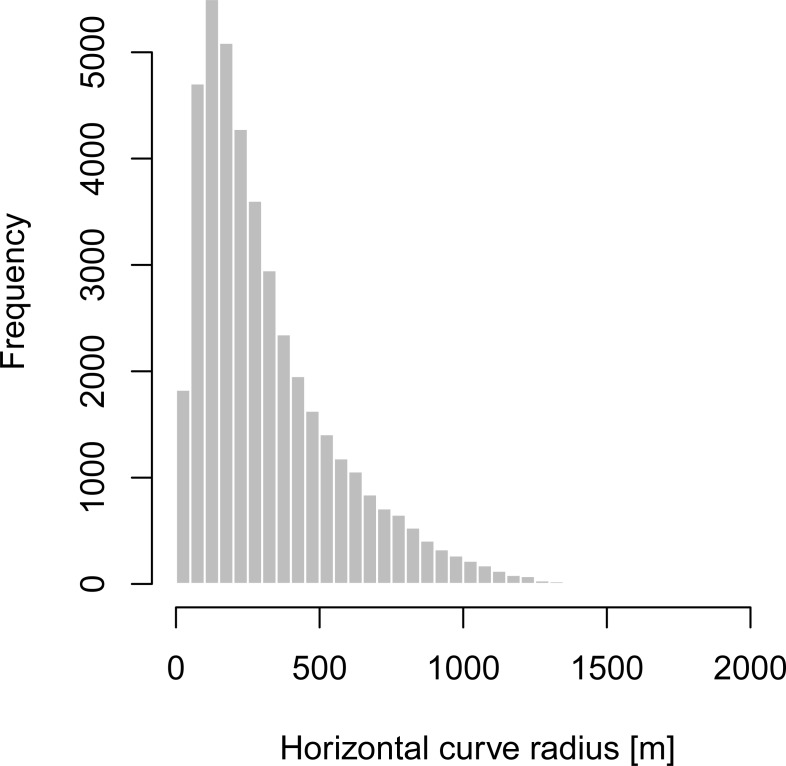
Distribution of radii for the secondary roads in the Czech Republic.

It was determined that approximately 43% (4,314 km) of the secondary roads within the Czech road network is formed by horizontal curves. In total, 42,752 horizontal curves were identified. All the results, including the segmented road network into the homogenous segments, can be seen at roca.cdvgis.cz/czechia web map application. As expected, the radius of 50–200 m is the most frequent. Slightly more than 40% of horizontal curves on the secondary roads have their radii lower than 200 m. Only 2% of horizontal curves on the secondary roads have their radii greater than 1000 m (Fig 5).

**Fig 6:**
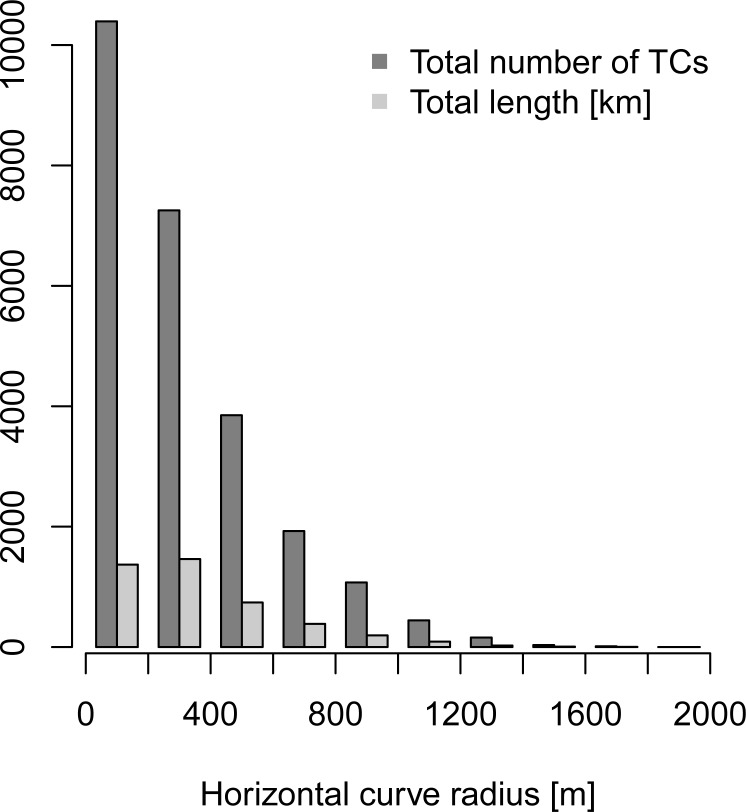
TCs which occurred on horizontal curves and the total lengths of horizontal curves with respect to the horizontal curve radius.

There were 56,710 traffic crashes (TCs) registered over the time period 2009–2016 which occurred on the secondary roads. In total, we considered 7,911 road sections. No crashes were reported at 18.1% of road sections (6.3% of the road network). We found out that 45.2% of TCs occurred at horizontal curves (see Fig 6 for their distribution with respect to the horizontal curve radius). Further information on the obtained results is summarized in Table 1.

**Fig 7:**
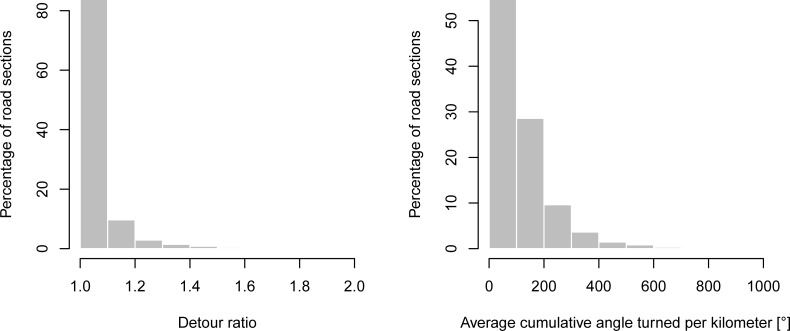
Distributions of the detour ratio (left) and the average cumulative angle turned per kilometer (right).

The detour ratio and average cumulative angle turned per kilometer were calculated for each road section as the output of the ROCA software. The detour ratio ranges from 1.0 (for a road section without any horizontal curve) to 6.0. Approximately 99% of road sections have detour ratios below 1.6 (see Fig 7). The average cumulative angle ranges from 0 to 5,044 degrees per kilometer with 95% values being lower than 642 degrees per kilometer (see Fig 7). The mean value and median of the average cumulative angle account for 224 and 94 degrees per kilometer, respectively.

### CMF for horizontal curves in the Czech Republic

After identifying the geometry of the secondary roads within the Czech road network, we developed a CMF for horizontal curves. First, we fitted model (5) by means of the negative-binomial regression. Regression coefficients and their 95% confidence intervals are provided in Table 2. We subsequently calculated the relative crash rates according to formula (6).

**Table 2:** Regression coefficients obtained from the negative-binomial regression along with their 95% confidence intervals (in brackets), and root-mean-square prediction error (RMSE) estimated by the use of 10-fold cross-validation.

	Coefficient (95% confidence interval)
*β*_1_	**-10.1** (-9.9, -10.4)
*β*_2_	**0.78** (0.92, 0.97)
*β*_3_	**0.88** (0.77, 0.87)
*β*_4_	**-0.15** (-0.21, -0.31)
*β*_5_	**0.32** (0.23, 0.40)
RMSE	1.58

**Fig 8:**
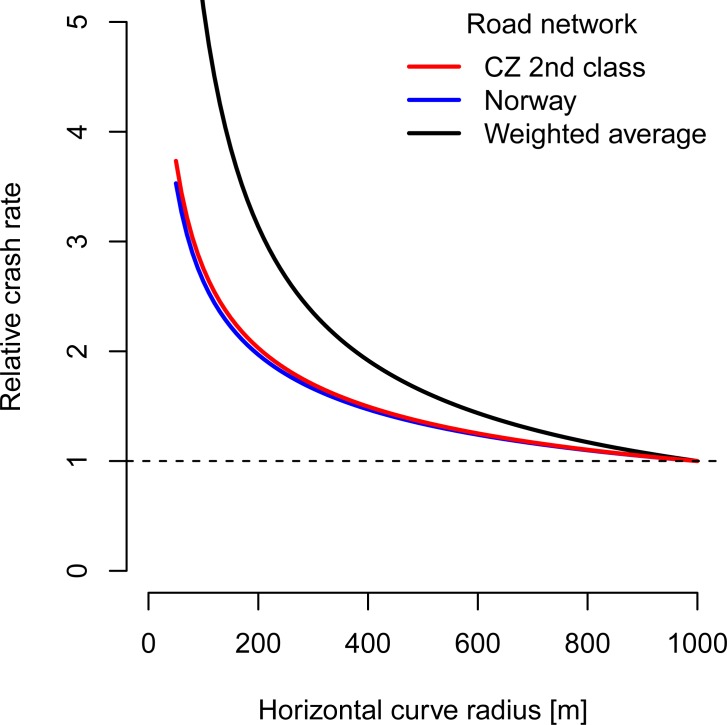
CMFs for horizontal curve radii on secondary roads in Czechia, in Norway [10] and the weighted average for eight countries calculated by Elvik [16].

Concerning CMF developed from the Czech road data for the secondary roads, the radius of the horizontal curve influenced the crash rate similarly as in the case of Norway (see Fig 8). Both these CMFs are much flatter than the summary CMF for eight countries developed by Elvik [16].

## Discussion and conclusion

The presented ROCA software allows for rapid road network data processing and identification of tangents and horizontal curves with their radii. It is able to significantly reduce the time of the traffic safety-related studies when road alignment is needed. The entire Czech road network analyzed into homogenous segments is accessible via roca.cdvgis.cz/czechia.

The ROCA software does not yet consider transition (spiral) curves connecting tangents and horizontal curves. The primary reason is the fact that the majority of data, currently stored in the databases, are manually digitized and therefore these segments with specific geometries are usually not represented in data, i.e. their identification is rather complicated.

The ROCA software allows users to define their own training data sets. This feature, when users are allowed to define geometry types with their own data, is extremely helpful as the national databases were created by means of various approaches and in various scales (resolution). The ROCA software can be downloaded for noncommercial application and research for free from roca.cdvinfo.cz.

It is worth mentioning that the performance of any software strongly depends on the quality of the input data. It is evident that precise results cannot be obtained when analyzing severely inaccurate data (see also [1]). We therefore encourage users to check the quality of the original road network data prior to the analyses.

### CMF for the Czech road network

We estimated a CMF for the secondary roads within the Czech road network to demonstrate the potential of ROCA SW. The horizontal curves with the lowest radii (50 m) are approximately 3.7 times more hazardous than curves with radii 1000 m. The results obtained in our study are in agreement with findings summarized by Elvik [16] who presented a metastudy on CMFs based on data from previously published studies from various countries. He concludes that *“a tendency is found for the accident rate to increase as the curve radius gets smaller”*. This is also valid for the secondary roads within the Czech road network (see Fig 8).
